# Comparative genomics and transcriptomics analysis of the bHLH gene family indicate their roles in regulating flavonoid biosynthesis in *Sophora flavescens*

**DOI:** 10.3389/fpls.2024.1445488

**Published:** 2024-09-24

**Authors:** Ake Liu, Junjie Lu, Huifang Song, Xi Wang, Mingyang Wang, Zhenhong Lei, Huixuan Liu, Haiying Lei, Tianzeng Niu

**Affiliations:** ^1^ Department of Life Sciences, Changzhi University, Changzhi, China; ^2^ School of Life Science, Shanxi Normal University, Taiyuan, China; ^3^ Shanxi Zhendong Pharmaceutical Co., Ltd., Changzhi, China

**Keywords:** bHLH, *Sophora flavescens*, gene duplication, flavonoid biosynthesis, coexpression network

## Abstract

The basic helix-loop-helix (bHLH) transcription factors play crucial roles in various processes, such as plant development, secondary metabolism, and response to biotic/abiotic stresses. *Sophora flavescens* is a widely used traditional herbal medicine in clinical practice, known for its abundant flavonoids as the main active compounds. However, there has been no comprehensive analysis of *S. flavescens bHLH* (*SfbHLH*) gene family reported currently. In this study, we identified 167 *SfbHLH* genes and classified them into 23 subfamilies based on comparative genomics and phylogenetic analysis. Furthermore, widespread duplications significantly contributed to the expansion of SfbHLH family. Notably, SfbHLH042 was found to occupy a central position in the bHLH protein-protein interaction network. Transcriptome analysis of four tissues (leaf, stem, root and flower) revealed that most *SfbHLH* genes exhibited high expression levels exclusively in specific tissues of *S. flavescens*. The integrated analysis of transcriptomics and metabolomics during pod development stages revealed that *SfbHLH042* may play a central role in connecting *SfbHLH* genes, flavonoids, and key enzymes involved in the biosynthesis pathway. Moreover, we also checked the expression of 8 *SfbHLH* genes using RT-qPCR analysis to realize the expression profiles of these genes among various tissues at different cultivated periods and root development. Our study would aid to understand the phylogeny and expression profile of SfbHLH family genes, and provide a promising candidate gene, *SfbHLH042*, for regulating the biosynthesis of flavonoids in *S. flavescens*.

## 1 Introduction

Transcription factors (TFs, also known as *trans*-acting factors) are a class of proteins that bind specifically to *cis*-acting elements in the promoter region of eukaryotic genes. They can regulate specific physiological or biochemical processes of cells at the transcriptional level and therefore function in different developmental stages of organisms (Feller et al., 2011; Gong et al., 2015). Since the first bHLH (basic helix-loop-helix) protein was found in rat (Murre et al., 1989), extensive studies showed that they are widely distributed in eukaryotes including fungi, animals and plants (Ledent and Vervoort, 2001; Jones, 2004). The bHLH domains usually have ~60 amino acids (AA) in eukaryotic organisms, consisting of two conserved regions. The basic region is located at the N-terminal of the domain and varies in length from 10-15 AA residues, mainly involved in the *cis*-element recognition and DNA binding. The HLH region is located at the C-terminal and consists of two α-helices containing hydrophobic residues and loop region with varying lengths (Massari and Murre, 2000; Toledo-Ortiz et al., 2003).

Accordingly, bHLH members were conserved among species, especially in bHLH domain. It has been reported that animal bHLHs were mainly classified into six primary groups (A-F group) based on their evolutionary relationships, dimerization ability and DNA-binding properties (Simionato et al., 2007). As one of the largest TF families in plants, bHLH genes were widely distributed and varied in numbers among all the plant lineages. Compared with animal bHLHs, more than 50% of plant bHLH members belong to group B, and 8% belong to group A (Nemie-Feyissa et al., 2015). The classifications of the bHLH gene family in plants is less clear than in animals due to its large variation. At present, the classifications of plant bHLHs mainly refer to the studies of Pires and Dolan (2010) and Carretero-Paulet et al. (2010). There were 26 bHLH subfamilies in former and 32 in latter when considering more ancestral plants, such as chlorophytes and algae. With more plant genomes released, many bHLH genes were identified and analyzed from different plants species, such as *Panax ginseng* (169 members), *Helianthus annuus* (183), *Andrographis paniculate* (122), *Pueraria lobata* (219), and etc (Chu et al., 2018; Li et al., 2021; Sun et al., 2022c; Xu et al., 2022; Xiao et al., 2023).

Accordingly, bHLH genes have diverse biological functions as a superfamily (Carretero-Paulet et al., 2010), such as regulating plant growth and development, abiotic stress and metabolic network response (Hewezi et al., 2006), as well as involved in the biosynthesis of plant secondary metabolites (Nemie-Feyissa et al., 2015; Wang et al., 2018). For instance, the maize *ZmLRL5* gene regulates root hair elongation and may affect the translational process or control the expression of ribosomal genes, which can make root hair growth sensitive to translational repression (Wang et al., 2019). Overexpression of *TabHLH39*, which is widely expressed across all the tissues of wheat, in *Arabidopsis thaliana* significantly enhanced plant tolerance to drought and low-temperature stresses (Zhai et al., 2016). The tobacco *NtbHLH123* enhances cold resistance by binding to the G-box/E-box element in the promoter regions of the tobacco *CBF* genes; in overexpressed plants, NtbHLH123 reduced cell membrane oxidative stress by decreasing hydrogen peroxide and reactive oxygen species accumulation from low temperatures (Zhao et al., 2018). The pepper CabHLH035 functions in salt tolerance by influencing intracellular Na^+^/K^+^ ratio and proline biosynthesis in *Capsicum* (Zhang et al., 2022). The apple MdbHLH3 enhances the cold resistance of apples by increasing anthocyanin accumulation at low temperature (Xie et al., 2012). The ZmbHLH55 increases the accumulation of ascorbic acid by directly regulating the gene expression related to ascorbic acid biosynthesis, thereby improving the salt tolerance of maize (Yu et al., 2021).

Moreover, bHLH transcription factors are also found involved in regulating the biosynthesis of secondary metabolites. Yeast one-hybrid assays showed that SmbHLH10 could directly activate the expression of enzyme genes in tanshinone biosynthesis pathway, thus increase the tanshinone accumulation in *Salvia miltiorrhiza* hairy roots (Xing et al., 2018). In *Paeonia suffruticosa*, PsbHLH1 can directly bind to the promoters of dihydroflavonol 4-reductase and anthocyanin synthetase genes to transcriptionally activate their expression and regulate the anthocyanin biosynthesis (Qi et al., 2020). In mulberry, *MabHLH3* is a key gene regulating the formation of fruit color, and its abnormal expression can disrupt the balance of anthocyanin regulatory network leading to the pigment composition differences (Li et al., 2020). As we know, improving secondary metabolites by increasing the expression of one or two enzyme genes is inefficient, whereas TFs can regulate the expression of multiple enzyme genes or even the entire metabolic pathway (De Boer et al., 2011). Thus, bHLH transcription factors are potential targets for studying plant secondary metabolism regulation and ideal candidate genes for molecular breeding.

*Sophora flavescens*, belong to the Leguminous family, is widely distributed in Asia (especially in the China, Korea and Japan) and some European countries (He et al., 2015). The roots of *S. flavescens*, known as “Kushen”, has been extensively used as traditional herbal medicine for their antipyretic, diuretic, and anthelmintic properties, as well as for the treatment of dysentery, gastrointestinal hemorrhage, vaginal itching and eczema (Lee et al., 2018). The main active compounds of *S. flavescens* are flavonoids and alkaloids. Recently, flavones from *S. flavescens*, especially isopentenyl flavones, have demonstrated promising potential in the fields of anticancer (Cheng et al., 2022), Parkinson’s disease relief (Sun et al., 2022a), and antibacterial activity (Li et al., 2022). The release of *S. flavescens* genome (Qu et al., 2023) provided us a valuable resource to determine the potential molecular mechanism for biosynthesis of its active compounds. In this study, we performed genome-wide identification, phylogenetic analysis, and their involvement in flavonoid biosynthesis. Our study would present a comprehensive analysis of *SfbHLH* genes and provide a reference for the further development and utilization of functional gene resources in medicinal herbs.

## 2 Results

### 2.1 Identification and sequence analysis of bHLH family members in *S. flavescens*

Totally, 167 putative *SfbHLH* genes were identified and renamed as *SfbHLH001* to *SfbHLH167* according to their physical positions on each chromosome (Chr, **Supplementary Figure S1**). It showed that the *SfbHLH* genes were irregularly distributed on 9 Chrs and 2 scaffolds. Among them, Chr3 had the highest density of bHLH genes (32), whereas Chr8 only 11 bHLH genes. As shown in **Supplementary Table S1**, the gene length of *SfbHLHs* varied from 600 bp (*SfbHLH022*) to 19472 bp (*SfbHLH005*), and their protein length ranged from 92 (SfbHLH134) to 813 (SfbHLH096) AA residues. The molecular weight (MW) of the bHLH proteins varied from 10.27 kDa (SfbHLH047) to 88.21 kDa (SfbHLH096), and their theoretical isoelectric points (pIs) were predicted to range from 4.48 (SfbHLH154) to 10.53 (SfbHLH093). We also identified 159 and 203 bHLH members from *Sophora moorcroftiana* and *Sophora japonica*. Compared to the well-studied bHLH genes from Arabidopsis and rice, *S. japonica* had the much more members and other two *Sophora* plants had similar counterparts.

**Figure 1:**
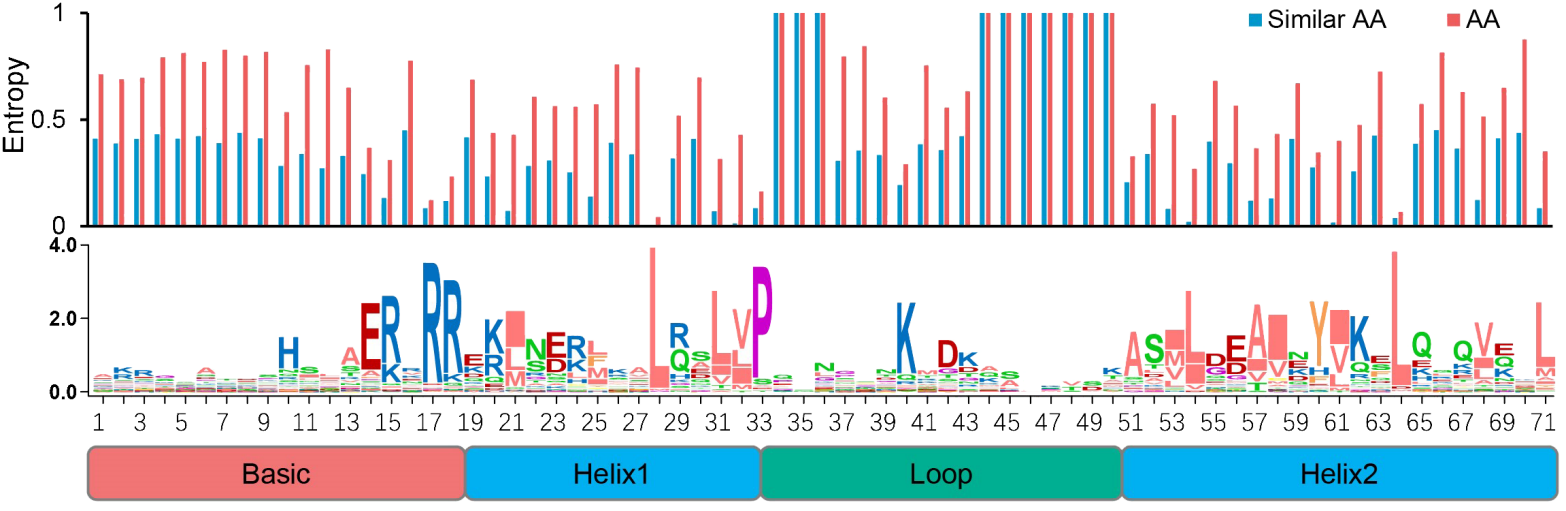
Conserved motif analysis of bHLH domain in *S. flavescens*. The overall height of each stack represents the conservation degree of the sequences at the position. The AA site with a conservation ratio more than 50% was identified with capital letters.

We also examined the conserved sites among the SfbHLH domains and found that 20 sites were conserved (**Figure 1**, with certain AA residue over 50% consensus ratio), among which six sites were highly conserved with a ratio more than 80% (R17, R18, L28, P33, K41, and L65). To ensure the AA conservation at different sites quantitatively rather than intuitively, we further calculated the AA entropy of each site within the bHLH domain. It showed that all the entropy values of above 20 conserved sites were less than 0.6, all of which were reported as conserved sites by previous study as well.

### 2.2 Phylogenetic analysis of *S. flavescens* bHLH members

**Figure 2:**
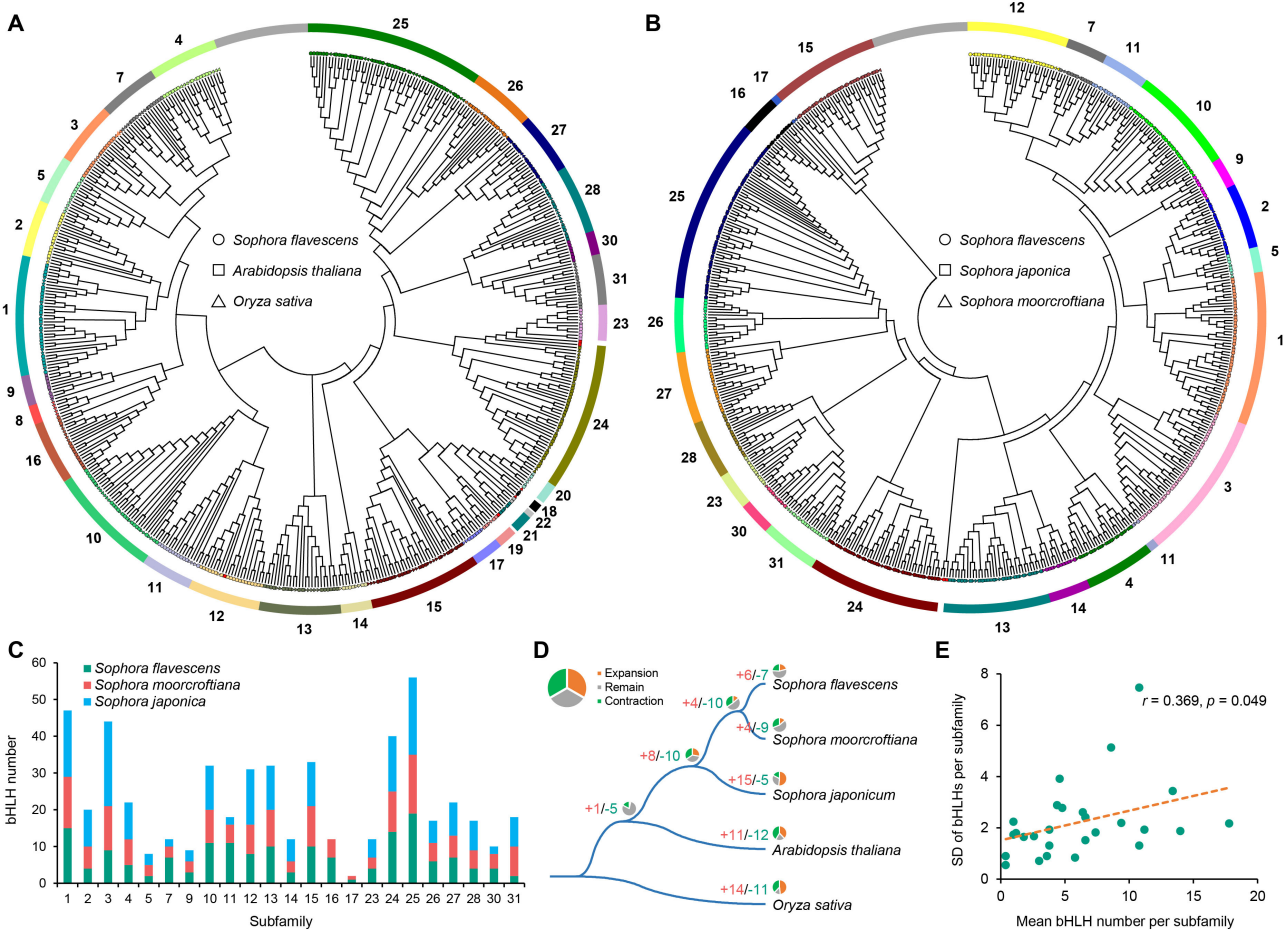
Evolutionary analysis of *bHLH* genes among five species. **(A)** Phylogenetic tree of bHLH proteins in *S. flavescens*, *A. thaliana* and rice; **(B)**. Phylogenetic tree of bHLH proteins in the *S. flavescens*, *S. moorcroftiana* and *S. japonica*; **(C)**. The number comparison of 23 subfamilies among the *S. flavescens*, *S. moorcroftiana* and *S. japonica*; **(D)**. Gene expansion and contraction among five species (CAFE 5); **(E)**. The standard deviations (SD) of bHLH genes per subfamily among five species are positively correlated with the average size of each subfamily.

According to the classification proposed by Carretero-Paulet et al. (2010), the 167 SfbHLH members were unevenly classified into 23 subfamilies (**Figure 2A**, subfamily 1-5, 7, 9-17, 23-28, 30 and 31) through phylogenetic analysis of SfbHLHs and the counterparts of *A. thaliana* and *O. sativa*. The subfamily sizes ranged from 2 to 19, with subfamily 25 having the largest size and subfamily 5 and 31 having the smallest one. Phylogenetic tree indicated that only few genes from these species exhibited one-to-one orthologous relationships, such as subfamily 10 (*SfbHLH107*, *OsbHLH053* and *AtbHLH098*), subfamily 15 (*SfbHLH103*, *OsbHLH071* and *AtbHLH110*), and subfamily 25 (*SfbHLH162*, *OsbHLH0095* and *AtbHLH048*) (**Figure 2A**; **Supplementary Figure S2**); more *bHLH* genes from above three species tended to cluster together independently in many subfamilies (subfamily 2, 3, 7, 9, 10, 11, 12, 13, 16, 24, 25, 27 and 30). It may imply that species-specific gene duplications tended to occur frequently after species divergence. For instance, subfamily 11 clustered into two clades. One only contained *OsbHLH147* and *SfbHLH0127*, and another contained 8 *SfbHLH* genes resulted from proximal duplication (PD) event (*SfbHLH050-057*) (**Supplementary Figure S1**).

To further understand the evolutionary relationship of the bHLH genes among the *Sophora* plants, we also conducted a phylogenetic analysis based on the alignment of *S. flavescens*, *S. moorcroftiana* and *S. japonica* bHLH domains. It subdivided these bHLHs unevenly into 23 well-defined subfamilies (**Figure 2B**; **Supplementary Figure S3**, subfamily 1-5, 7, 9-17, 23-28, 30 and 31). The size of these subfamilies ranged from 2 to 19, with subfamily 25 having the largest size and subfamily 17 having the smallest one. The subfamily 17 only contained 2 bHLH members (*S. flavescens* and *S. moorcroftiana* each containing one bHLH gene). The bHLH members were relatively close in the subfamilies between *S. flavescens* and *S. moorcroftiana* (**Figure 2C**). Moreover, we conducted an analysis of the gene structure and motif distribution of the *SfbHLH* genes (**Supplementary Figure S4**), which showed that genes belonging to the same subfamily tend to have similar characteristics, providing evidence for the subfamily classification based on evolutionary analysis. We further conducted subfamily expansion and contraction of bHLH genes among five species, which showed that *S. flavescens* is more similar to *S. moorcroftiana*, with only a few subfamily members experienced either expansion or contraction (**Figure 2D**, subfamily expansion: 6 and 4; contraction: 7 and 9). While 15 bHLH subfamilies expanded, and 5 contracted in *S. japonica*. Hence, the evolutionary path of *S. japonica* tends to enter a relatively independent branch diverged from *S. flavescens* and *S. moorcroftiana*.

Moreover, the differences of gene numbers in each subfamily indicated that the subfamily 8 varied the most (coefficient of variation (CV) = 69.8%), whereas subfamily 19 varied the least (CV = 13.6%). The standard deviations (SD) of the bHLH subfamily size among these three species were significantly positively correlated with the mean numbers of bHLH genes in each subfamily (Pearson’s correlation coefficient (*r*) = 0.369, *p* < 0.05; **Figure 2E**). This means that smaller sized subfamilies were generally more conserved across species, but larger ones tend to be more variable.

### 2.3 Duplications contributing to the expansion of *S. flavescens* bHLH family

**Figure 3:**
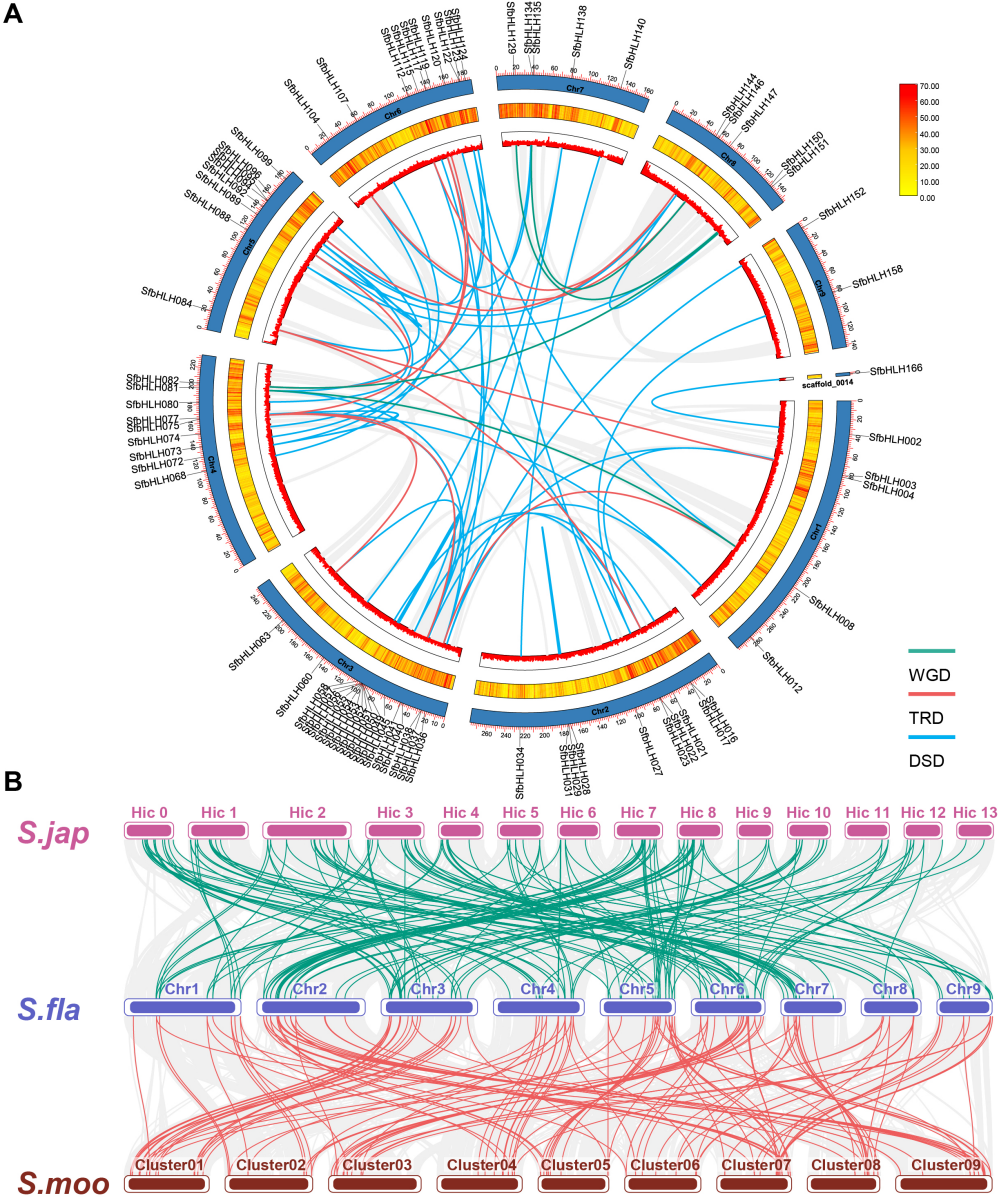
Gene duplication pattern and syntenic analyses of bHLH genes in *S. flavescens*, *S. moorcroftiana* and *S. japonica*. **(A)**. The locations of each *SfbHLH* gene on 9 chromosomes and 1 scaffold. To avoid crowding, we only show the names of these *SfbHLH* genes experienced WGD, TRD and DSD events on the Circos diagram. The different colors (red to yellow) in the middle track indicated high to low gene density. **(B)**. The collinear relationship between *S. flavescens*, *S. moorcroftiana*, and *S. japonica*. The collinear blocks are represented by gray lines, while the *bHLH* gene collinearity is indicated by green and red lines. *S. fla*, *S. moo*, and *S. jap* were short for *S. flavescens*, *S. moorcroftiana*, and *S. japonica*, respectively.

Gene duplication contributes greatly to the amplification of gene family during the evolution. Here, we identified the potential gene duplication events among bHLH family in *S. flavescens* using DupGen_finder (**Figure 3A**). It showed that 4 gene pairs were derived from whole genome duplication (WGD, *SfbHLH008* vs *-081*, *-082* vs *-151*, *-129* vs *-150*, and *-135* vs *-147*), 11 gene pairs were derived from transposed duplication (TRD, *SbHLH004* vs *-084*, *-021* vs *-084*, *-038* vs *-008*, *-041* vs *-075*, *-063* vs *-075*, *-089* vs *-082*, *-096* vs *-144*, *-104* vs *-147*, *-112* vs *-144*, *-117* vs *-075*, and *-119* vs *-075*). Moreover, one tandem duplication (TD) gene pair (*SbHLH122* vs *-123*), and three PD pairs (*SfbHLH016* vs *-017, -035* vs *-036*, and *-159* vs *-160*) and one clusters (*SfbHLH050-057*) were identified (**Supplementary Figure S1**). Additionally, 48 gene pairs were originated from dispersed duplication (DSD). Totally, 76 *SfbHLHs* may result from duplication events. Except the duplicated gene pairs (*SbHLH002*/*-166* and *-074*/*-094*), the *Ka*/*Ks* ratios of any two paralogous genes ranged from 0.105 to 0.883, indicating that they were subjected to purifying selection (**Supplementary Table S2**). Hence, PD, TRD and DSD contributed greatly to the expansion of *S. flavescens* bHLH family.

To further address the potential evolutionary patterns of the SfbHLH gene family and the phylogenetic relationship, a comparative orthologous analysis was performed between *S. flavescens* and the other two *Sophora* plants (*S. moorcroftiana* and *S. japonica*). The orthologous gene pairs between *S. flavescens* and *S. moorcroftiana* and *S. japonica* were 120 and 109, respectively. Synteny analysis reveals that the identified orthologous genes of *SfbHLH*-*SmbHLH* were more than those of *SfbHLH*-*SjbHLH* (**Figure 3B**). Hence, *S. flavescens* exhibited a higher level of collinearity with *S. moorcroftiana* than *S. japonica*.

### 2.4 Protein interaction network and function enrichment analysis

**Figure 4:**
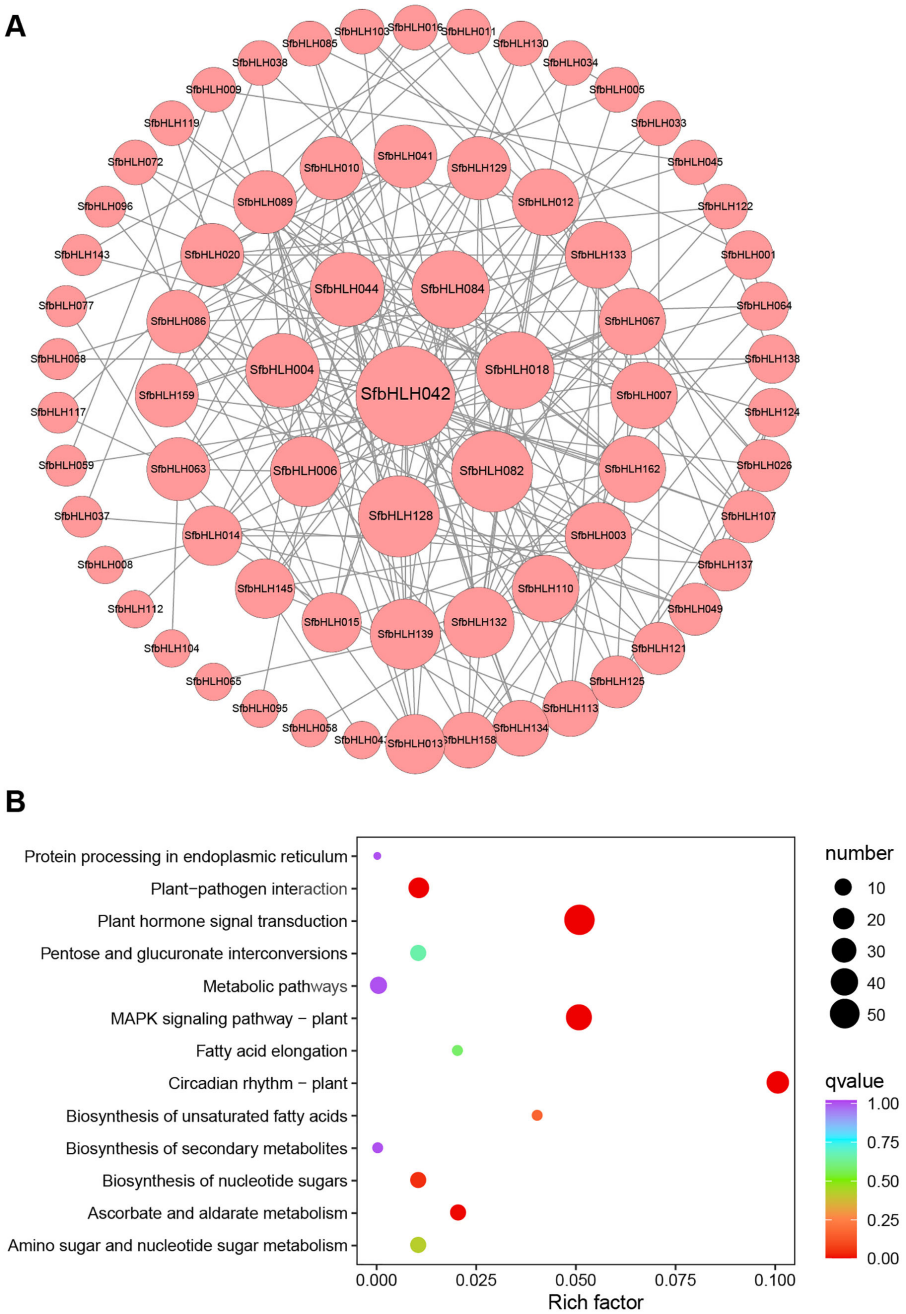
Protein-protein network and function analysis of SfbHLHs. **(A)** Protein-protein network for SfbHLHs constructed according to information available about Arabidopsis bHLHs in STRING database through orthologous relationship. **(B)**. KEGG enrichment analysis of *SfbHLH* genes. The size of the dot indicates the bHLH number in certain pathway, and the color reflects the *p* value.

Different bHLH proteins can form homodimers or heterodimers thus to bind DNA and regulate the transcription of downstream targets. Therefore, protein–protein interaction (PPI) is fundamentally important for understanding protein functions. The results showed that a total number of 70 nodes with 201 edges are included in the network (**Figure 4A**), and majority SfbHLHs interacted with more than one bHLH protein. The proteins interact most frequently among SfbHLH042, -082, -128, -084, -018, -044, -004, etc, and are more centrally located (edge > 10). We also performed KEGG pathway analysis for 167 *SfbHLH* genes, and screened 8 significantly enriched pathways (**Figure 4B**, *p* < 0.05), including plant circadian rhythm, plant hormone signal transduction, MAPK signaling pathway-plant, plant-pathogen interaction, ascorbate and aldarate metabolism, biosynthesis of nucleotide sugars, biosynthesis of unsaturated fatty acids, and amino sugar and nucleotide sugar metabolism.

### 2.5 Expression patterns of *S. flavescens bHLH* genes among different tissues

**Figure 5:**
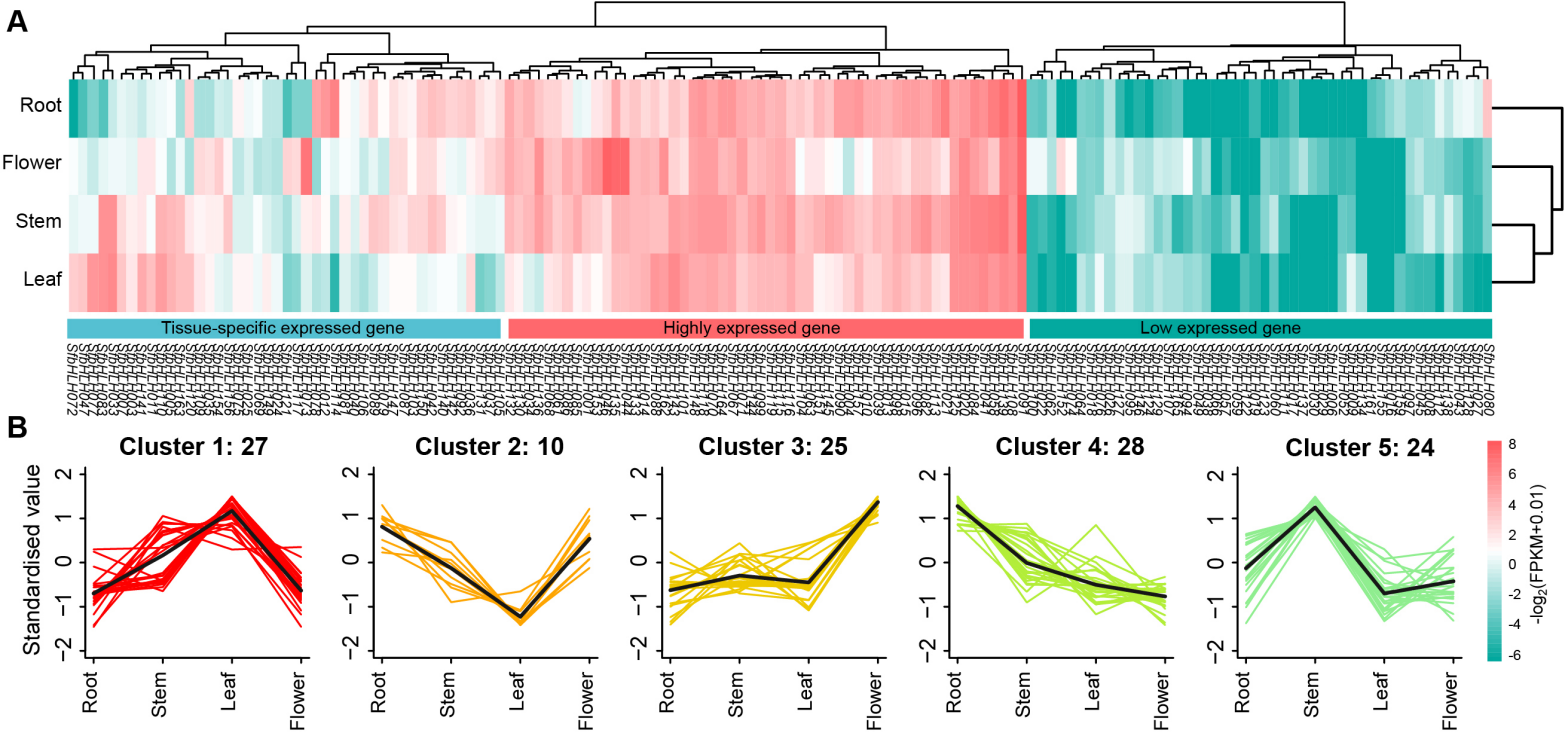
Expression patterns of SfbHLH genes among different tissues. (**A)** The expression profile of *SfbHLHs* among four tissues, including root, stem, leaf and flower. (**B)**. K-means cluster analysis of the expression of *SfbHLHs* among four tissues. Those *SfbHLHs* with expression values less than 1 in all the four tissues were excluded from the cluster analysis.

To determine whether the family members are tissue-specific or constitutionally expressed, we analyzed the expression levels of the SfbHLH members among four tissues in *S. flavescens* (**Figure 5A**, flower, leaf, stem and root). The expression of *SfbHLH* genes was variable and mainly divided into three patterns: 68 *SfbHLH* genes tend to be low expressed across the four tissues (including 20 *SfbHLH* genes not detected expression); 54 *SfbHLH* genes tend to be highly expressed across the four tissues; and 45 *SfbHLH* genes tend to be tissue-specific expressed. Majority *SfbHLH* genes were identified to be expressed in the roots. K-means cluster analysis showed five gene clusters: the relative expression level of *SfbHLH* genes in cluster 1 (27 genes) was the highest in leaves but those in cluster 2 (10) lowest in leaves, respectively; in cluster 3 (25), the expression in flowers was highest and that of other three tissues was relatively lower; the expression in stems was highest in cluster 5 (24), while that in roots was highest in cluster 4 (28) (**Figure 5B**). The above results indicated that most *SfbHLH* genes tend to be only highly expressed in certain tissue of *S. flavescens*.

### 2.6 Expression comparison of *SfbHLH* genes during the pod development

**Figure 6:**
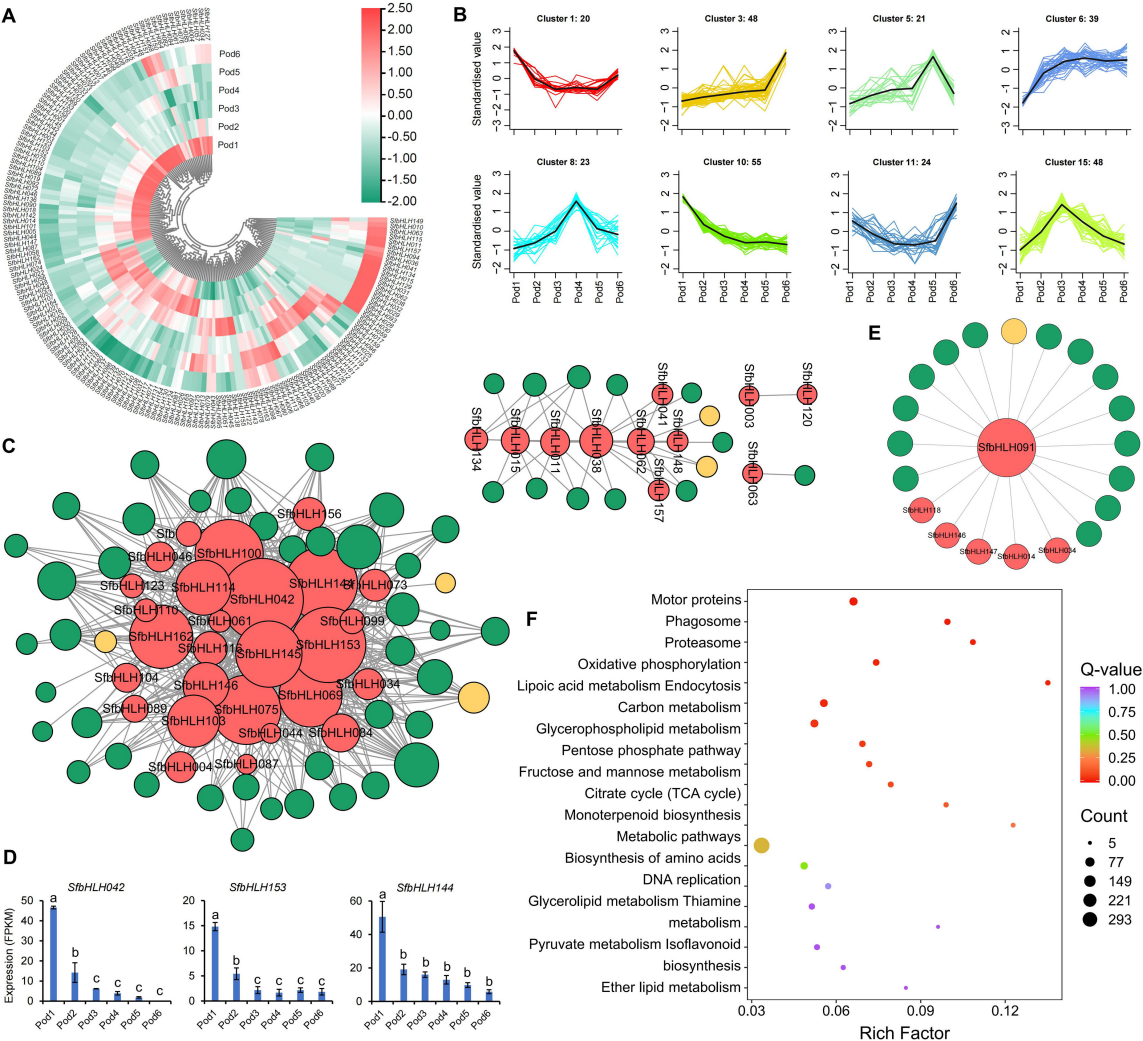
Expression profiles of the *S. flavescens* pods at different developmental periods. **(A)**. The expression profile of *SfbHLHs* during the pod development. **(B)**. K-means cluster analysis of the expression of *SfbHLHs* and key enzymes in the flavonoid synthesis pathway, and the accumulation of the *S. flavescens* flavonoids during the pod development. **(C)**. The co-expression network of *SfbHLHs*, key enzymes and flavonoids. The red dot represents the *SfbHLH* gene, the green dot represents flavonoid metabolite, and the yellow dot represents key enzyme in the flavonoid synthesis pathway. **(D)**. The expression of hub *SfbHLH* genes during the pod development. **(E)**. The co-expression network of *SfbHLH091*, key enzymes and flavonoids. **(F)**. The KEGG enrichment analysis of genes co-expressed with *SfbHLH091*.

We conducted analysis of the *SfbHLH* expression profiles during the *S. flavescens* pod development. Among them, Pod1 and Pod6 had most *SfbHLH* genes with relative higher expression levels, while Pod2 also had more *SfbHLH* genes highly expressed (**Figure 6A**). Among all the *SfbHLH* genes in *S. flavescens*, only *SfbHLH091* displayed a high level of expression in each tissue or development stage, while *SfbHLH002* and *-126* was not expressed across the tested samples. The K-means clustering analysis (**Figure 6B**) revealed that the cluster 1 and 11 showed that the expression level of *SfbHLH* genes was at a higher level in Pod1 and Pod6, the expression level tended to decrease from the stage of Pod2, and it was at a smoother level to Pod5. These *SfbHLH* genes in cluster 3 are slowly upregulated in the first five stages (Pod1~5), and their expression rises sharply to a maximum in the sixth stage (Pod6), which may function in the accumulation of metabolites. The genes from cluster 5, 8, and 15 all showed up-regulated and then down-regulated, which may regulate the biosynthesis of metabolites.

The co-expression network of *SfbHLH* genes with the *S. flavescens* flavonoids and enzymes showed that the genes, *SfbHLH153*, *-042*, *-144*, and *-145*, acted as hub-genes (**Figure 6C**), implying their key roles in the regulating biosynthesis of flavonoids during pod development. The key enzyme in the most highly interconnected flavonoid metabolic pathway within the network was FG2 (flavonol-3-O-glucoside L-rhamnosyltransferase), which exhibited the highest degree of connectivity (12). The secondary metabolites, Isohyperoside, Quercetin-3-O-glucoside (isoquercitrin), and Tricin-7-O-Glucuronide, exhibited the highest level of connectivity, with edges of 20, 25, and 25, respectively. We further individually analyzed the expression of three genes with top edge number in the network, *SfbHLH042*, *-153* and *-144*, at different developmental stages of pods, to find that the expression levels of these three genes had similar trends (**Figure 6D**). At Pod1 stage, the expression level of three *SfbHLHs* was the highest, while at Pod2 stage, its expression level was doubly reduced. The expression level of *SfbHLHs* was then slowly decreased at Pod3, Pod4 and Pod5 stage until it was minimized at Pod6 stage. Meanwhile, we analyzed the co-expression of *SfbHLH091* with 5 other SfbHLH genes and 1 flavonoid enzyme gene (*I2′H*). Additionally, we found that 14 flavonoid compounds (**Figure 6E**) had the strongest connection with *SfbHLH091*. Furthermore, a functional enrichment analysis revealed significant enrichment of these co-expressed genes in 9 KEGG pathways, including motor proteins, phagosome, proteasome, oxidative phosphorylation, lipoic acid metabolism, endocytosis, carbon metabolism, and glycerophospholipid metabolism (**Figure 6F**).

### 2.7 Expression comparison of *SfbHLH* genes during the root development and in different tissues with different cultivated years

**Figure 7:**
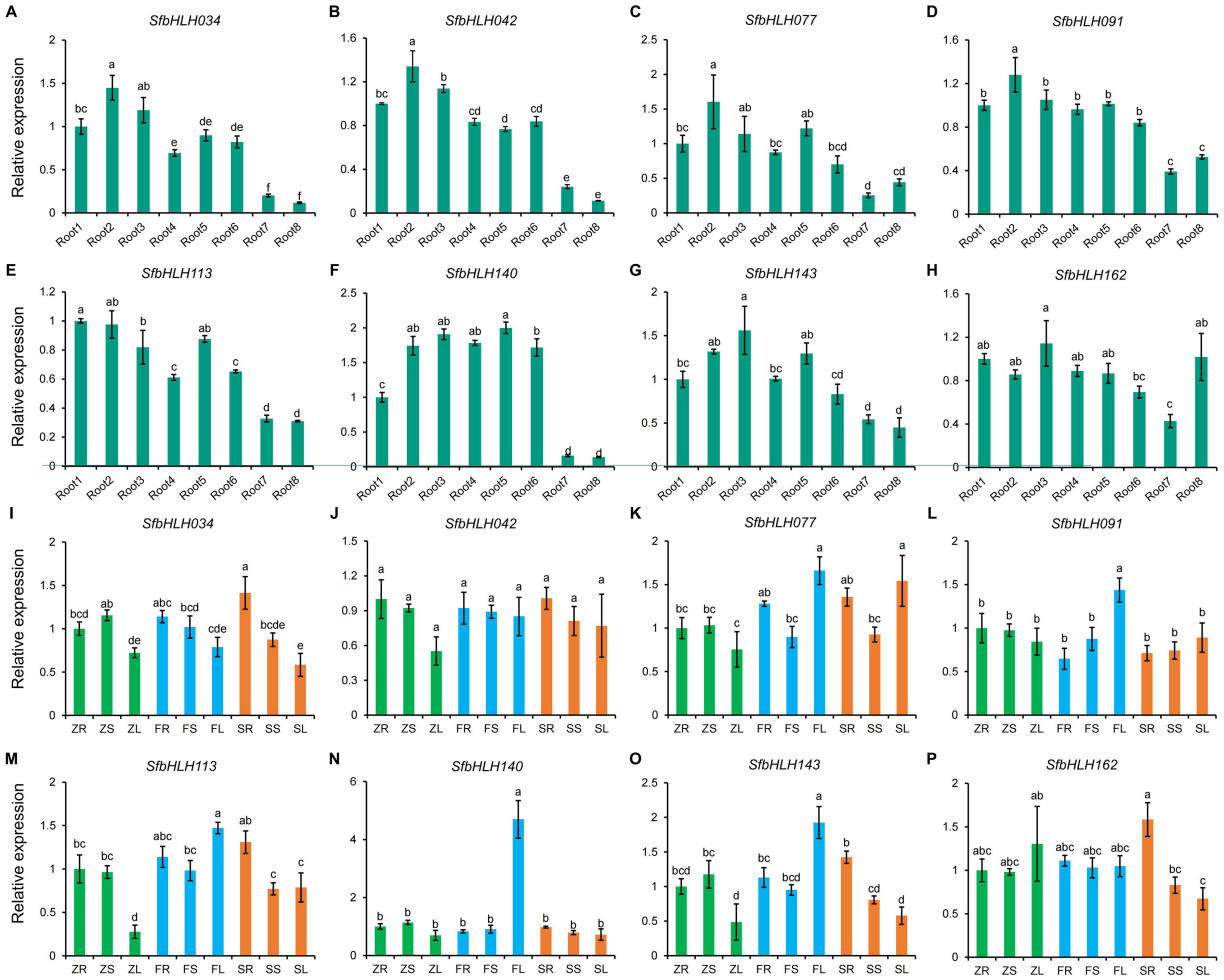
Expression analysis of *SfbHLH* genes during the root development and among different tissues with different cultivated years by RT-qPCR. **(A–H)**. The expression levels of different *SfbHLH* genes during the root development. **(I–P)**. The expression levels of different *SfbHLH* genes among different tissues with different cultivated years. Each sample contain three biological replicates. Different letters on different columns indicate a significant difference between any two samples (*p* < 0.05).

To further detect the *SfbHLH* genes during the root development stages, we selected 8 genes (*SfbHLH034*, *-042*, *-077*, *-091*, *-113*, *-140*, *-143* and *-116*) with high expression levels. The expression patterns of *SfbHLH034*, *-042*, *-077*, *-091*, *-140*, and *-143* were highly similar and their expression levels in roots showed a trend of first increase and then decrease during the root development of *S. flavescens* (**Figures 7A–H**). Moreover, the expression patterns of 8 SfbHLH genes were also analyzed by qRT-PCR assay among different tissues (root, stem and leaf) from zero-year plants (Z), first-year plants (F), and second-year plants (S) (**Figures 7I–P**). The results showed that 8 *SfbHLHs* expressed among all the tissues. Compared with other genes, the expression level of *SfbHLH042* was more constant in all the tissues tested and the *SfbHLH042* showed highest level in S roots (SR). *SfbHLH162* was expressed at relatively higher levels in Z leaves (ZL) and S roots (SR). Additionally, the expression levels of 8 *SfbHLHs* in the stems across different cultivated period kept basically unchanged. In summary, spatiotemporal expression analysis results indicated that 8 *SfbHLH* genes exhibited various expression patterns, which provide preliminary information for understanding their potential functions in *S. flavescens*.

## 3 Discussion

*S. flavescens* is an important medicinal herb, whose root has a long history in the application of Chinese traditional medicine (Sun et al., 2022b). Given the abundant flavonoids present in the root (He et al., 2015), it is imperative to investigate the potential regulatory mechanism underlying its biosynthesis in *S. flavescens*. Accordingly, *bHLH* genes are involved in regulating flavonoid biosynthesis in medical plants (Zhu et al., 2020; Xiao et al., 2023). However, there has been no report to systematically carry out comprehensive identification of bHLH members in *S. flavescens*.

Based on sequence homology and phylogenetic relationships, higher plants such as *A. thaliana*, *O. sativa*, and sunflower have been identified to encode a large number of bHLH family genes (Carretero-Paulet et al., 2010; Li et al., 2021); while some lower plants have fewer bHLH family genes (Merchant et al., 2007; Rensing et al., 2008). In this study, 167 *SfbHLH* genes were identified in *S. flavescens* genome (**Figure 1**), which were subdivided into 23 subfamilies (**Figure 2A**). The variation in the numbers of *bHLH* genes among species might be related to gene duplication events rather than the genome size (Flagel and Wendel, 2009). The number of *bHLH* genes differed among closed related species *S. flavescens*, *S. moorcroftiana* and *S. japonica*, possibly due to species-specific duplication events.

Gene duplication patterns usually reveal their generation and evolutionary changes in function (Wang et al., 2020). Our results indicated that the expansion of *SfbHLH* genes may be driven by duplications. Although *S. flavescens* was undergone two rounds of WGD events (Qu et al., 2023), only four WGD pairs were identified (**Figure 3A**). However, many other duplication patterns have been identified, such as 48 DSD and 11 TRD pairs of *SfbHLH* genes. Strong functional constrain makes duplicate *bHLHs* undergo purifying selection (Flagel and Wendel, 2009). Our results indicate that TRD and DSD play critical roles in the expansion of the bHLH gene family in *S. flavescens*, leading to potential subfunctionalization or neofunctionalization, as well as alterations in gene expression patterns and protein-protein interactions. Synteny analysis of *S. flavescens*, *S. moorcroftiana* and *S. japonica* suggests a specific expansion of *SfbHLHs* in *S. flavescens*.

Currently, the biological functions of most *SfbHLHs* remain elusive. However, leveraging comparative genomics enables us to predict gene functions in *S. flavescens* through ortholog analysis with extensively studied species. For example, the bHLHs that are currently known to regulate flavonoid biosynthesis belong to subgroup IIIf and II(d+e) (corresponding subfamily 5 and 2 in our study) (Feller et al., 2011). To date, numerous bHLH proteins have been identified to form complex with other family proteins, playing a crucial role in the regulation of flavonoids biosynthesis. For instance, the *VvMYC1* gene encoding bHLH transcription factor interacts with MYB to regulate the expression of three flavonoids biosynthetic genes, including *ANR*, *UFGT*, and *CHI* (Hichri et al., 2010). Arabidopsis CPC (MYB) and GL3 (bHLH) can affect the anthocyanin biosynthesis in tomato (Moriguchi et al., 2014). It was notable known that MBW protein complex (bHLH, WD40, and MYB proteins) can regulate flavonoids biosynthesis (Baudry et al., 2004; Baudry et al., 2006; Gonzalez et al., 2007).

The functions of *bHLH* genes are diverse, such as the development of organs and tissues, biosynthesis and metabolism of secondary metabolites (Pires and Dolan, 2010). The transcriptome data of *S. flavescens* were further scrutinized to provide insight into the potential functions. Our study systematically analyzed the transcriptomic pattern of *SfbHLH* genes from different tissues or different growth stage in *S. flavescens* and found significant correlations between several *SfbHLH* genes and flavonoid content, implying their involvement in the flavonoid biosynthesis of *S. flavescens*. Accordingly, conducting co-expression analysis on gene family members is of great significance in deeply understanding their regulatory functions (Bano et al., 2021a; Bano et al., 2021b; Bano et al., 2023). Therefore, our study conducted co-expression analysis and functional enrichment analysis of SfbHLH family members with other genes based on transcriptome data, which will provide reference for understanding the functions of these family members (**Figure 6**). However, further experimental information is needed to validate this conclusion, such as transgenic functional verification in *S. flavescens*.

## 4 Conclusion

We totally identified 167 *SfbHLH* genes and classified them into 23 subfamilies based on phylogenetic analysis. There were 76 *SfbHLHs* may result from gene duplication events, especially proximal duplications, transposed duplications, and dispersed duplications, which significantly contribute to the expansion of the *S. flavescens* bHLH family. Notably, SfbHLH042 was found to occupy a central position in the bHLH protein-protein interaction network and the network connecting *SfbHLH* genes, flavonoids, and key enzymes involved in the flavonoid biosynthesis pathway. RT-qPCR analysis also exhibited the expression profiles of some *SfbHLH* genes during root development and among three tissues with different cultivated years. Our study may provide reference information for the further function study of *SfbHLH* genes and insight into the molecular mechanism of flavonoid biosynthesis in *S. flavescens*.

## 5 Materials and methods

### 5.1 bHLH gene identification and sequence retrieval in *S. flavescens*

The *S. flavescens* genome sequences and annotation files were obtained from Qu et al. (2023). To determine the potential SfbHLH sequences encoding in *S. flavescens* genome, the entire *S. flavescens* protein sequences were searched by the HMMER3.4 software (http://hmmer.org/) using an *E*-value threshold of 10^−2^ (Prakash et al., 2017), and the sequences containing the HLH domains (PF00010) identified by HMM profile from Pfam database (http://pfam.xfam.org) (El-Gebali et al., 2019) were kept. For those genes with alternative splicing, we preserved only the longest protein sequence for each gene. Both SMART domain analysis tool (http://smart.embl.de/) (Letunic and Bork, 2018) and bHLH consensus (allowing 13 mismatches from the plant bHLH consensus) (Carretero-Paulet et al., 2010) was used to check whether containing the typical domain architecture. BLAST searches were carried out using putative SfbHLH and *A. thaliana* bHLH sequences to ensure the integrity of the determined bHLHs (*E*-value of 10^−8^). Moreover, the *bHLH* genes from *S. moorcroftiana* (Yin et al., 2023), and *S. japonica* (Lei et al., 2022) were identified using the same method with *S. flavescens*. The *bHLH* genes from other plants, including *A. thaliana* and *Oryza sativa* (rice), were obtained from previous study (Carretero-Paulet et al., 2010). In addition, proteins with incomplete or non-typical HLH domains were manually excluded from this study.

### 5.2 Chromosomal location, sequence characterization and syntenic analysis

To obtain the physical location of the SfbHLH repertoire on the chromosomes, the 167 *SfbHLHs* were mapped to the 9 chromosomes and two scaffolds of *S. flavescens* by TBtools (v1.0692) (Chen et al., 2020). The SfbHLHs were named based on their physical location on each chromosome or scaffold from top to bottom. To detect the protein properties, the MWs and pIs were predicted by the ProtParam program (ExPASy tools, http://web.expasy.org/protparam/) on the basis of all the high-confidence SfbHLH sequences. CELLO v2.5 tool (http://cello.life.nctu.edu.tw) was used to predict subcellular localization of bHLHs (Yu et al., 2006). The exon-intron organizations for structural diversity of *S. flavescens* bHLH genes were identified through comparing their predicted coding sequences (CDSs) based on the genome annotations (Yu et al., 2006). The Multiple Em for Motif Elicitation 5.0.5 (MEME, http://meme-suite.org/) online program was used to annotate the conserved motifs along these proteins (Bailey et al., 2009). The classification of bHLH DNA-binding motifs were identified according to their basic regions of the bHLH domain following the description in the previous study (Yu et al., 2006). The final graphical presentation of motif distribution and gene structure was performed by TBtools (v1.0692) (Chen et al., 2020).

To determine the duplication patterns of the *SfbHLH* genes encoding in the *S. flavescens* genome, DupGen_finder was used to identify WGD, TD, PD, TRD and DSD (Qiao et al., 2019). The syntenic analysis of *SfbHLHs* was performed by the Multiple Collinearity Scan toolkit (Mcscanx) for the detection of *bHLH* gene pairs duplication events within *S. flavescens* genome, with default parameters (*E*-value of 10^-10^) (Wang et al., 2012). Finally, non-synonymous (*Ka*) and synonymous (*Ks*) substitution rates between *SfbHLH* genes were calculated using KaKs_Calculator 3.0 to select YN (Yang and Nielsen) models (Zhang, 2022).

### 5.3 Multiple sequence alignment and phylogenetic analysis

The sequences of *S. flavescens*, *A. thaliana* and rice bHLH proteins, as well as three leguminous plant *S. flavescens*, *S. moorcroftiana* and *S. japonica* were applied to perform multiple sequence alignment with bHLH domain sequences using ClustalW program build-in MEGA 7 (v7.0.26) with default setting, respectively (Kumar et al., 2016). A neighbor joining (NJ) phylogenetic tree was reconstructed using Jones-Taylor-Thornton (JTT) model based on the above alignment with 1000 bootstrap replicates, pairwise deletion, and other default parameters of MEGA 7. Subfamily classification of SfbHLH proteins was determined according to Carretero-Paulet et al. (2010).

### 5.4 Construction of protein interaction networks and functional enrichment analysis

The functional PPI networks were generated with STRING (https://string-db.org) using the protein sequences of the SfbHLH family to search against the Arabidopsis databases. The PPI Networks were visualized using Cytoscape (v.3.8.2) (Otasek et al., 2019). Functional enrichment analyses of genes, including GO and KEGG analyses, were carried out using the Metware Metabolic Cloud tool (https://cloud.metware.cn).

### 5.5 Expression profile of *SfbHLH* genes in different tissues and periods

To study the expression patterns of *SfbHLH* genes in different tissues and grow period, we collected 4 different tissues (stem, flower, leaf and root) and 6 different growth period of pods for RNA-seq from more than 5 years *S. flavescens* cultivated in Changzhi International Shennong Traditional Chinese Medicine Cultural Expo Park (Shangdang District, Changzhi City, Shanxi Province). The pods were collected from July 12, 2021 to Augest 6, 2021 (Samples were taken every five days). We also collected 3 different tissues (stem, leaf and root) *S. flavescens* with different cultivated years and 8 different growth period of roots used for quantitative real-time PCR (RT‒qPCR) from *S. flavescens* cultivated in Dianshang County (Lucheng District, Changzhi City, Shanxi Province). Among the different cultivated years, Z indicated *S. flavescens* seeds sowned in 2024, F represented those sowned in 2023, and S denoted those sowned in 2022. The *S. flavescens* seeds are typically sowned in April each year, and the leaves (L), strems (S) and roots (R) were collected in July, 2024. The samples of root development were collected from *S. flavescens* (sowned in April, 2022) on the 20th of each month from April to November, 2023. Each sample with three biological replicates was collected in a 50 mL centrifugal tube and immediately put into liquid nitrogen for quick freezing, and then removed and stored in -80°C for later use.

### 5.6 WGCNA analysis

The experimental methods and analysis pipeline of the transcriptome (stem, flower, leaf and root, and pod development) and metabolome (pod development) were following Zhong et al. (2024). The raw data of RNA-seq samples was deposited in NCBI database (the accession NO. listed in **Supplementary Table S3**). Gene co-expression network was constructed using WGCNA (v1.71) (Langfelder and Horvath, 2008) implemented in R (v 4.2.2) for the combined *S. flavescens* transcriptome and metabolome data set of pod transcriptome. A soft threshold (β) of 18 was chosen to make the network present an approximately scale-free topology. Then, the neighbor-joining matrix was converted to a topological overlap matrix (TOM) to calculate the corresponding degree of dissimilarity. A hierarchical clustering module is constructed using the dissimilarity based on the TOM values, and a clustering tree diagram is generated based on the TOM. In the co-expression network, the edge weights (weight values, ranging from 0 to 1, corresponding to the interaction strength) of any two connected genes were determined based on their topological overlap metric. Networks with inter-gene weight values greater than 0.3 were visualized using Cytoscape.

### 5.7 Expression of *SfbHLHs* during root development and among three tissues with different cultivated years through RT-qPCR

The RT‒qPCR analysis for *SfbHLH* genes during root development and among three tissues with different cultivated years was conducted as follow. Total RNA of 17 *S. flavescens* samples with three biological replicates was extracted using polysaccharide polyphenol plant total RNA extraction kit (Beijing GeneBetter, China). After confirming the integrity of the RNA using Nanodrop 2000 (Thermo Fisher, USA), reverse transcription was carried out using HiScript II Q RT SuperMix (Vazyme, Nanjing), and qPCR was performed using SYBR qPCR Master Mix (Vazyme, Nanjing). Gene-specific primers were designed using Primer5 software (v5.00); the primer sequences are shown in **Supplementary Table S4**. *EF-1α* was used as the reference gene. All reactions were carried out in three replicates, and gene expression levels were calculated by 2^−△△Ct^. All statistical analyses and significance tests were performed using SPSS based on one-way analysis of variance (ANOVA) and Duncan’s test.

## Data Availability

The datasets presented in this study can be found in online repositories. The names of the repository/repositories and accession number(s) can be found below: https://www.ncbi.nlm.nih.gov/, PRJNA1136989.

## Supplementary material

Supplementary Figure 1 Chromosomal locations of *S. flavescens bHLH* genes. The name of *SfbHLH* genes is assigned according to their physical position of the chromosomes.

Supplementary Figure 2 The NJ tree was constructed based on the alignment of bHLH domains in the *S. flavescens*, *A. thaliana* and rice. The boostrap numbers on the nodes can support the reliability of topology.

Supplementary Figure 3 The NJ tree was constructed based on the alignment of bHLH domains in the *S. flavescens*, *S. moorcroftiana* and *S. japonica*.

Supplementary Figure 4 The gene structure and motif distribution of bHLH members in the *S. flavescens*.

Supplementary Table 1 Detailed information of the *S. flavescens*, *S. moorcroftiana*, and *S. japonica* bHLH gene family.

Supplementary Table 2 Synonymous and nonsynonymous substitution rates for the duplicated gene pairs among *S. flavescens* bHLH genes. *Ka*, nonsynonymous substitution rate; *Ks*, synonymous substitution rate.

Supplementary Table 3 The accession NO. of RNA-seq raw data.

Supplementary Table 4 Primer sequences used for RT‒qPCR.
