# Supplementary figures and images for: Comparative genomics and transcriptomics analysis of the bHLH gene family indicate their roles in regulating flavonoid biosynthesis in Sophora flavescens
Source: Front Plant Sci. 2024 Sep 24;15:1445488. doi: 10.3389/fpls.2024.1445488 (PMC11458398; doi:10.3389/fpls.2024.1445488)

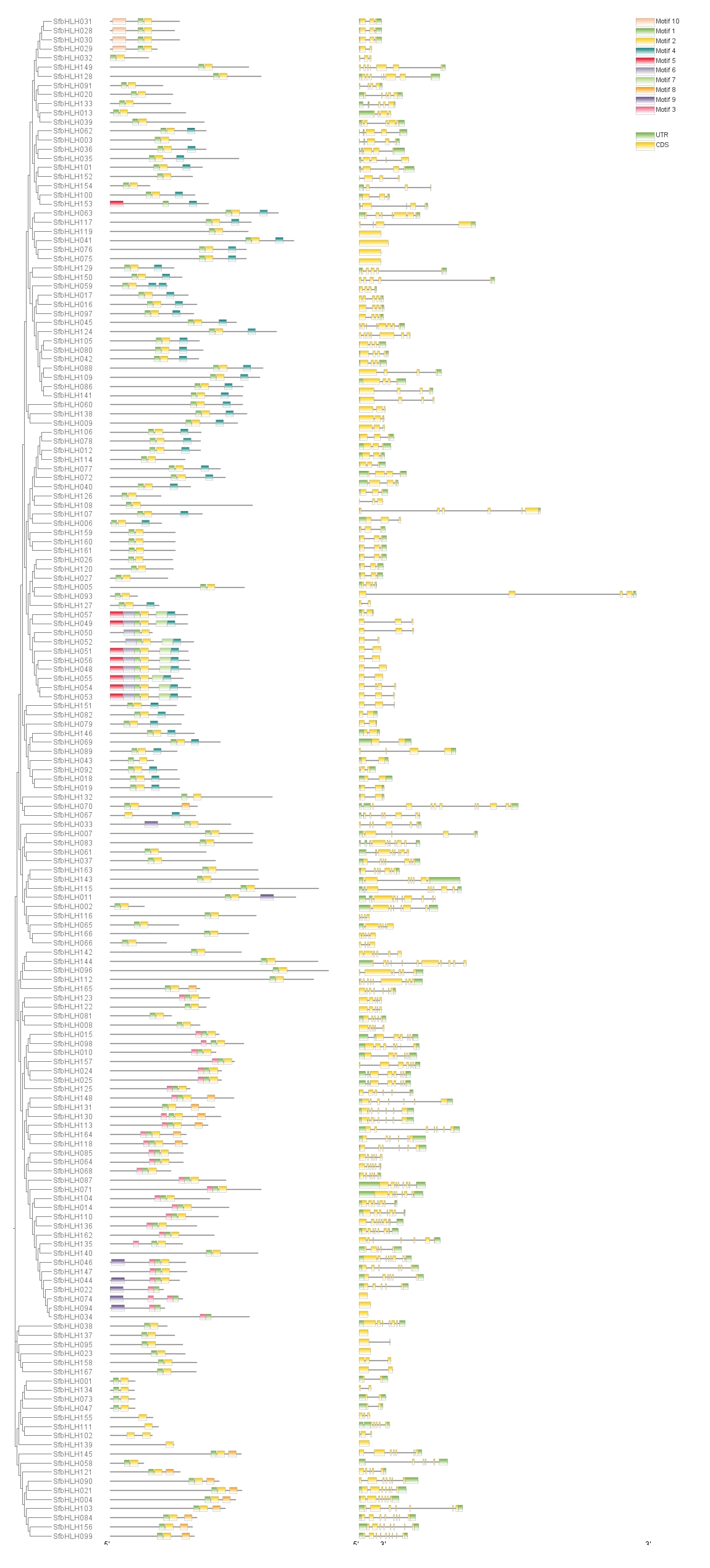

Supplement: Supplementary Figure 1 — Chromosomal locations of S. flavescens bHLH genes. The name of SfbHLH genes is assigned according to their physical position of the chromosomes. [file Image1.jpeg]

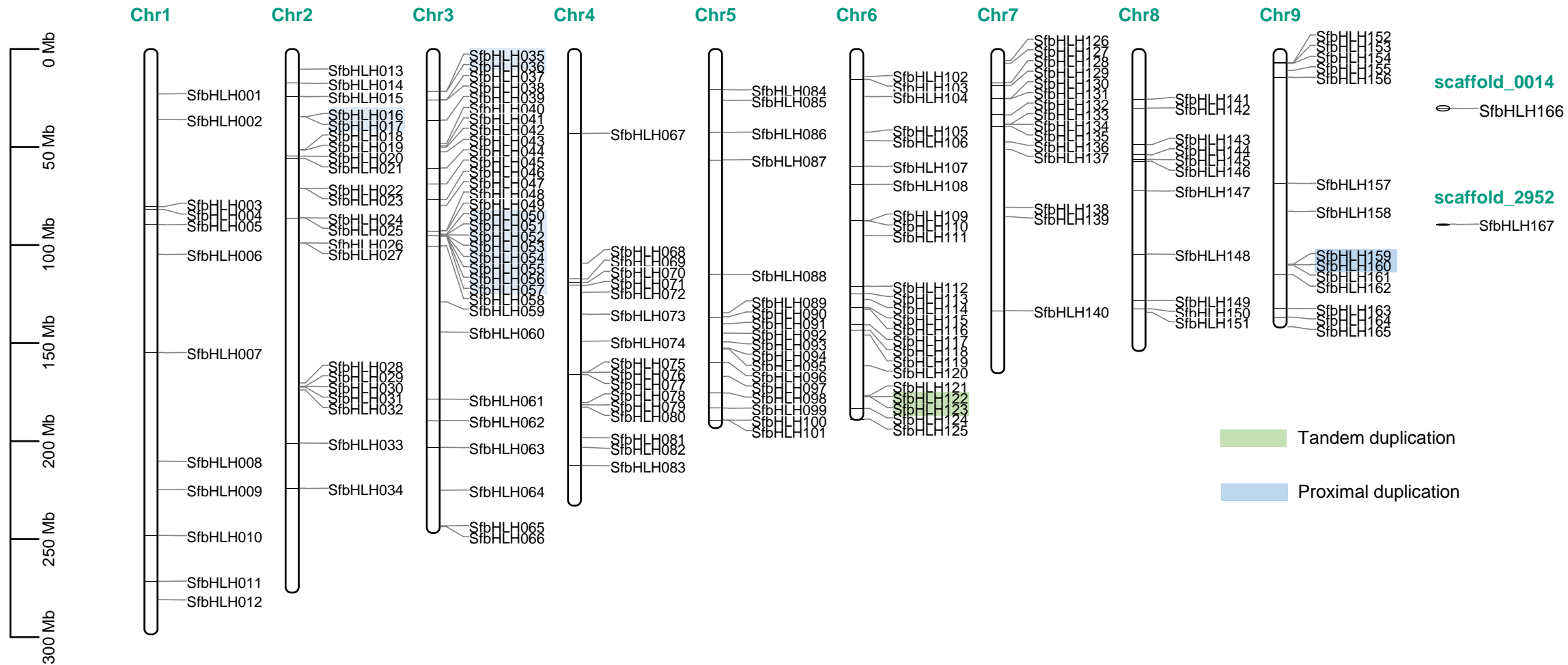

Supplement: Supplementary Figure 2 — The NJ tree was constructed based on the alignment of bHLH domains in the S. flavescens, A. thaliana and rice. The boostrap numbers on the nodes can support the reliability of topology. [file DataSheet1.pdf]

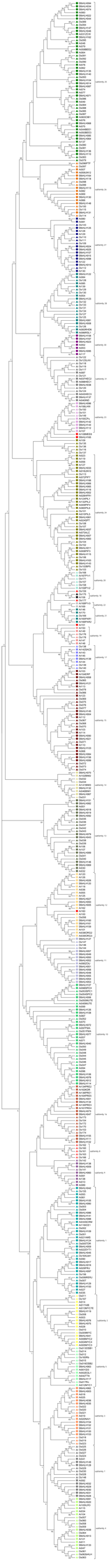

Supplement: Supplementary Figure 3 — The NJ tree was constructed based on the alignment of bHLH domains in the S. flavescens, S. moorcroftiana and S. japonica. [file DataSheet2.pdf]

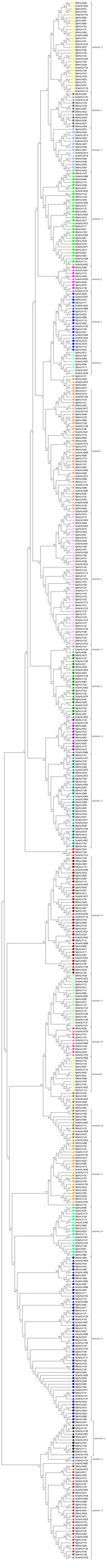

Supplement: Supplementary Figure 4 — The gene structure and motif distribution of bHLH members in the S. flavescens. [file DataSheet3.pdf]
